# Psychometric Properties of the Problematic Internet Use Questionnaire Short-Form (PIUQ-SF-6) in a Nationally Representative Sample of Adolescents

**DOI:** 10.1371/journal.pone.0159409

**Published:** 2016-08-09

**Authors:** Zsolt Demetrovics, Orsolya Király, Beatrix Koronczai, Mark D. Griffiths, Katalin Nagygyörgy, Zsuzsanna Elekes, Domokos Tamás, Bernadette Kun, Gyöngyi Kökönyei, Róbert Urbán

**Affiliations:** 1 Institute of Psychology, Eötvös Loránd University, Budapest, Hungary; 2 Psychology Division, Nottingham Trent University, Nottingham, United Kingdom; 3 Institute of Sociology and Social Policy, Corvinus University of Budapest, Budapest, Hungary; 4 ECHO Sociological Research Institute, Budapest, Hungary; University of Ariel, ISRAEL

## Abstract

Despite the large number of measurement tools developed to assess problematic Internet use, numerous studies use measures with only modest investigation into their psychometric properties. The goal of the present study was to validate the short (6-item) version of the Problematic Internet Use Questionnaire (PIUQ) on a nationally representative adolescent sample (n = 5,005; mean age 16.4 years, SD = 0.87) and to determine a statistically established cut-off value. Data were collected within the framework of the European School Survey Project on Alcohol and Other Drugs project. Results showed an acceptable fit of the original three-factor structure to the data. In addition, a MIMIC model was carried out to justify the need for three distinct factors. The sample was divided into users at-risk of problematic Internet use and those with no-risk using a latent profile analysis. Two latent classes were obtained with 14.4% of adolescents belonging to the at-risk group. Concurrent and convergent validity were tested by comparing the two groups across a number of variables (i.e., time spent online, academic achievement, self-esteem, depressive symptoms, and preferred online activities). Using the at-risk latent profile analysis class as the gold standard, a cut-off value of 15 (out of 30) was suggested based on sensitivity and specificity analyses. In conclusion, the brief version of the (6-item) PIUQ also appears to be an appropriate measure to differentiate between Internet users at risk of developing problematic Internet use and those not at risk. Furthermore, due to its brevity, the shortened PIUQ is advantageous to utilize within large-scale surveys assessing many different behaviors and/or constructs by reducing the overall number of survey questions, and as a consequence, likely increasing completion rates.

## Introduction

Problematic Internet use and Internet addiction became a widely researched area since the first scientific papers on the topic [1–4]. A number of more recent studies have focused on the development of measurement instruments to assess the problem [5, 6]. However, many of these used convenience samples, did not undergo a thorough validation process, and/or featured ad-hoc cut-off points [7].

In a previous analysis, Koronczai and colleagues [8] suggested that a suitable measure for assessing problematic online use should meet six basic requirements. The measure should be (i) *comprehensive*, examining more (possibly all) aspects of problematic internet use; (ii) as *concise as possible*, in order to be able to assess the more impulsive population, and to fit into time-limited surveys (or having different forms with different length), (iii) reliable and valid for *different methods of data collection* (e.g. online, paper-and-pencil self-rating, face-to-face); (iv) appropriate for *different age groups* (e.g. adolescents and adults), and (v) appropriate in different *cultural* settings, and (vi) incorporate cut-off scores defined on the basis of clinical examination.

The Problematic Internet Use Questionnaire (PIUQ) [9] fulfills several of the six criteria. It is a comprehensive measure assessing three basic aspects of problematic Internet use: obsession (i.e., obsessive thinking about the Internet and, mental withdrawal symptoms caused by the lack of Internet use), neglect (i.e., neglect of basic needs and everyday activities) and control disorder (i.e., difficulties in controlling Internet use). It has two versions (18-item and 9-item), both having reliable factor structures, and proven validity across both online and paper-pencil data collection methods on samples of different age groups (i.e., adults and adolescents) [8, 9].

The experience of large-scale surveys internationally has demonstrated that using concise and brief instruments is critical in reducing the overall number of survey questions and likely increasing completion rates and help avoiding survey fatigue [10]. Therefore the goal was to create an even shorter (6-item) version of the PIUQ and validate it on a nationally representative sample of adolescents. A further aim was to determine a statistically and empirically well-established cut-off value for diagnosing problematic Internet use.

## Materials and Methods

### Participants and procedure

A nationwide adolescent sample was collected within the framework of the European School Survey Project on Alcohol and Other Drugs (ESPAD) project [11]. This international project comprising 36 European countries collects data on smoking, alcohol, and drug use from adolescents aged 16 years. In addition to the mandatory questions, each country had the opportunity to include optional questions. In 2011, Hungary included a brief additional section to assess problematic Internet use.

To obtain a representative sample of the 16 years old Hungarian population, three grades (8–10) were included in the sample, each grade containing a proportion of the target population. An internationally homogenous stratified random sampling method was applied based on region (Central/Western/Eastern Hungary), grade (8–10), and class type (primary general, secondary general, secondary vocational, and vocational classes). The sampling unit was the class, and every student who was at school at the time of data collection completed the questionnaire. Data were weighted due to a refusal rate of 15% causing a skewed nonresponse. To match the composition of the participants with the sampling frame, data were weighted by strata, with the matrix weighting method according to the National Education Information System (KIR-STAT). Questions regarding problematic Internet use were only included for the representative sample of 9th–10th graders in secondary general and secondary vocational schools (n = 5,112). After removing cases where problematic Internet use questions were completely missing, the final sample size was 5,005 participants.

The study design was based on an international protocol approved by the European School Survey Project on Alcohol and Other Drugs (ESPAD) Assembly, which was conducted in full compliance with the principles expressed in the Declaration of Helsinki. Written informed consent was obtained from both the students and their parents (on behalf of their children). As per the legacy of the institutional review board of the Corvinus University of Budapest, the affiliation of the leading researcher (Zsuzsanna Elekes) responsible for the data collection of the present study, survey studies such as the current one can be carried out without additional ethical permissions, solely based on the approval of the international ESPAD Assembly. Consequently, the present study has met the ethical requirements of the institutional review board of the Corvinus University of Budapest.

### Measures

Major socio-demographic characteristics such as age, gender, grade, residence, and Internet use habits (i.e., duration of a typical daily Internet session, type of Internet activities pursued) were collected. Additionally, psychological characteristics, including self-esteem (the Hungarian version of the Rosenberg’s Self-Esteem Scale [RSES-HU] [12, 13]) and depressive mood (short-form [6-item] Center of Epidemiological Studies Depression-Scale [CES-D] [14]) were assessed. RSES is a self-report unidimensional measure of global self-esteem assessing feelings of self-worth and self-acceptance. It has 10-items (five reversed) that are answered on a 4-point scale (‘‘strongly agree” to ‘‘strongly disagree”). Scores range from 10 to 40, with higher scores indicating higher self-esteem. Cronbach’s alpha on the present sample was 0.86. Short-form CES-D is also a unidimensional scale designed to assess depressive symptom levels. The instrument was translated and then back-translated by Hungarian experts in the addiction field. The back-translation was then compared to the original instrument and adjustments were made. The 6 items are answered on a 4-point scale (‘‘rarely or never” to ‘‘most of the time”). Scores range from 6 to 24, with higher scores indicating higher depressive mood level. Cronbach’s alpha on the present sample was 0.82.

Problematic Internet use was measured using the 6-item version of the PIUQ (see S1 Appendix). The PIUQ was originally an 18-item scale with good psychometric properties, originally developed on a Hungarian sample and later on validated on different samples with different data collection methods and used in several studies in various countries [8, 9, 15–17]. As noted above, it assesses three dimensions of problematic Internet use (i.e., obsession, neglect, and control disorder). The 6-item version of PIUQ was developed by selecting two items from each of the three factors. Item selection took into account preservation of high content validity and selection of the highest possible factor loadings. Participants use a 5-point Likert scale (from “never” to “always/almost always”) to estimate how much the given statement characterized them. Scores range from 6 to 30, with higher scores indicating increased problematic use. The primary reason for developing a short scale was to create a measure brief enough to assess more impulsive populations in time-limited surveys.

### Statistical analysis

A confirmatory factor analysis was applied to confirm the measurement model of problematic Internet use. Due to serious deviation from normal distribution (left skewedness), responses to the six items were treated as ordinal variables and the robust weighted least square (WLSMV) estimation method was used as recommended by Brown [18] and Muthén and Muthén [19]. Two nested measurement models were compared using a chi-square difference test, the originally proposed three-factor model, and an alternative one-factor structure. The latter was included due to high correlations (0.80–0.94) between the three subscales.

To evaluate the model fit, multiple indices were selected, including the chi-square (χ^2^) value, the comparative fit index (CFI), the Tucker–Lewis Fit index (TLI), the root mean square error of approximation (RMSEA), and its 90% confidence interval (90% CI), and p value smaller than 0.05 for test of close fit (Cfit..05). The chi-square test has to be non-significant (*p* > .05) for a good fit. However, this index is sensitive to large sample sizes, therefore alternative indices are used in addition. CFI and TLI values larger than 0.95 indicate an excellent fit, and values larger than 0.90 indicate an acceptable fit [20]. An RMSEA value below 0.05 indicates excellent fit, while a value above 0.1 signals a poor fit [18].

To test the discriminant validity of the three factors, a multiple indicators-multiple causes (MIMIC) analysis with WLSMV estimation method was applied. The MIMIC technique, a specification of structural equation modeling, was chosen for the present study because MIMIC models can estimate the effect of indicators on latent variables at the same time when direct effects of grouping variables or other continuous variables on the latent variables are also included. MIMIC modeling also helps clarify the construct validity of the three PIUQ factors.

Since there is no gold standard to determine problematic Internet use, a latent profile analysis (LPA) was carried out to identify those users whose Internet use was considered as problematic. LPA is a mixture modeling technique used to identify groups of people (categorical latent variables) who give similar responses to specific variables [21], in the present case scores given to the three PIUQ factors (continuous manifest variables). The analysis was carried out with one to three classes. To determine the number of latent classes, multiple indices were used: the measures of parsimony (i.e., Akaike Information Criteria [AIC], Bayesian Information Criteria [BIC], and Sample size adjusted Bayesian Information Criteria [SSABIC]) with lower values indicating more parsimonious models, the Entropy criterion that determines the accuracy of classifying people into their respective profiles (higher values indicating better fit), and the Lo-Mendell-Rubin Adjusted Likelihood Ratio Test (LMRT). The LMRT statistically compares the fit of the tested model (e.g., three-class model) to a model with one class less (e.g., two-class model). A significant *p* value (< .05) indicates that the tested model fits better than the previous one [19].

To test the criterion validity of the PIUQ-SF-6, the LPA groups were compared across variables related to problematic Internet use (i.e., gender, age, daily Internet use, grade point average, self-esteem, level of depressive symptoms, and preferred online activities). For these comparisons, a Wald’s chi-square test of mean equality was used for latent class predictors in mixture modeling because it takes into consideration the probabilistic nature of the LPA classes (for description of analysis, see www.statmodel.com/download/meantest2.pdf). All analyses were conducted on the weighted sample. Missing data were treated with Full-information maximum likelihood (FIML) method [22]. Statistical analyses were carried out with Mplus 6.0 [22] and IBM SPSS Statistics for Windows, Version 20.0 [23].

## Results

### Descriptive statistics

The mean age of the sample was 16.4 years (SD = 0.87) with age ranging from 15 to 23 years. (The wide age range was due to a small number of older students [approx. 1.5% of the sample] still attending the 9^th^ or 10^th^ grades at the age of 19 years or older in the time of data collection.) Approximately half of the sample (50.8%) was male. Two-thirds of the adolescents (68.8%) used the Internet in their disposable leisure time almost every day, and an additional 22.5% used the Internet at least once a week. Using social networking sites was among the top three Internet activities for 77.7% of the participants, followed by online chatting (68.4%), downloading content (e.g., music, movies) (57.8%), online searching for information (45.5%), playing online games (23.8%), and sending emails (18.0%).

### Confirmatory factor analysis

The three-factor solution recommended during the development of the instrument [6, 7] was tested with confirmatory factor analysis (CFA). The model provided an acceptable fit to the data (χ^2^ = 212.4 df = 6 *p* < .001; CFI = 0.983; TLI = 0.957; RMSEA = 0.083 [0.074–0.093]). Alternatively the model fit of the unidimensional model (χ^2^ = 412.9 df = 9 *p* < .001; CFI = 0.966; TLI = 0.943; RMSEA = 0.095 [0.087–0.103]) was tested. The original three-factor model yielded superior fit compared to the one-factor model (χ^2^-test = 194.1; Δdf = 3, *p* < .0001). In the three-factor model, the factor loadings were between .54 and .86 (see Table 1).

**Table 1:** Standardized estimates of factor loadings of three-factor solution for each item of Problematic Internet Use Questionnaire Short Form (PIUQ-SF-6).

	Obsession	Neglect	Control disorder
2. How often do you feel tense, irritated, or stressed if you cannot use the Internet for as long as you want to?	.82		
6. How often does it happen to you that you feel depressed, moody, or nervous when you are not on the Internet and these feelings stop once you are back online?	.86		
1. How often do you spend time online when you’d rather sleep?		.54	
5. How often do people in your life complain about spending too much time online?		.72	
3. How often does it happen to you that you wish to decrease the amount of time spent online but you do not succeed?			.73
4. How often do you try to conceal the amount of time spent online?			.79
Mean	1.53	2.10	1.55
Standard deviation	0.77	0.87	0.76
Obsession		.87	.80
Neglect			.94
Control disorder			

Empty cells represent the factor loadings that are fixed to 0; all other factor loadings are significant at least at *p* < .001. Cronbach’s alpha of the Problematic Internet Use Questionnaire Short Form is .77. N = 5005.

In order to further test the need for three distinct factors, a MIMIC analysis was carried out. The three factors were compared along variables relevant to problematic Internet use (see Table 2). The MIMIC model showed adequate fit to the data (χ^2^ = 453.9 df = 42 *p* < .001; CFI = 0.955; TLI = 0.906, RMSEA = 0.047 [0.043–0.050]). Results show that a considerable part of these variables influence the three factors differently (i.e., gender, daily internet use, grade point average, and preferred online activities).

**Table 2:** MIMIC model to test discriminant validity of the three factors.

Predictor variables	Obsession	Neglect	Control disorder
Gender	-.04	-.14***	-.04
Age	-.02	-.02	-.05*
Internet use in an average day (≥6 hours)	.21***	.31***	.08***
Grade point average	.01	.07***	.09***
Self-esteem	-.08***	-.07**	-.11***
Level of depressive symptoms	.32***	.32***	.31***
Top three Internet activities:			
Surfing, browsing, searching for information	-.06**	< .01	.01
Playing online games	.11***	.13***	.08***
Online chatting, talking (e.g., *MSN*, *Skype*)	.06**	.20***	.07**
Social networking (e.g., *Facebook*, *Twitter*)	.04*	.13***	.07**
Sending e-mails	-.05*	-.07**	-.02
Downloading (e.g., music, movies)	-.02	.04*	-.02
*R*^*2*^	.22	.33	.17

Standardized regression coefficients are presented.

*p < .05

**p < .01

***p < .001.

*R*^*2*^: Explained variance of latent factors. Gender was coded as 1 for male and 2 for female. N = 4526 due to missing values in the predictor variables.

### Latent profile analysis

A latent profile analysis with one to three classes was performed on the three dimensions of problematic internet use. The fit indices and test values are presented in Table 3. According to the criteria listed in the ‘Statistical analyses’ section the two-class solution was selected. The AIC, BIC, and SSABIC decreased continuously as more classes were added. However, the degree of decrease diminished after the third class was added. Regarding the entropy, the two-class solution reached the greatest value, and the examination of the L-M-R test values and their level of significance clearly showed that the three-class solution should be rejected in favor of the two-class solution.

**Table 3:** Fit Indices for the Latent Profile Analysis of the Problematic Internet Use Questionnaire Short Form (PIUQ-SF-6).

*Number of latent classes*	*AIC*	*BIC*	*SSABIC*	*Entropy*	*L-M-R test*	*p value*
1	56190	56229	56210			
2	51879	51945	51913	0.912	4196	.0003
3	50552	50643	50599	0.898	1297	.1760

AIC, Akaike Information Criteria; BIC, Bayesian Information Criteria; SSABIC, sample size adjusted Bayesian Information Criteria. LM-R test, Lo-Mendell-Rubin adjusted likelihood ratio test value; *p*, *p* value associated with L-M-R test. N = 4994.

**Fig 1:**
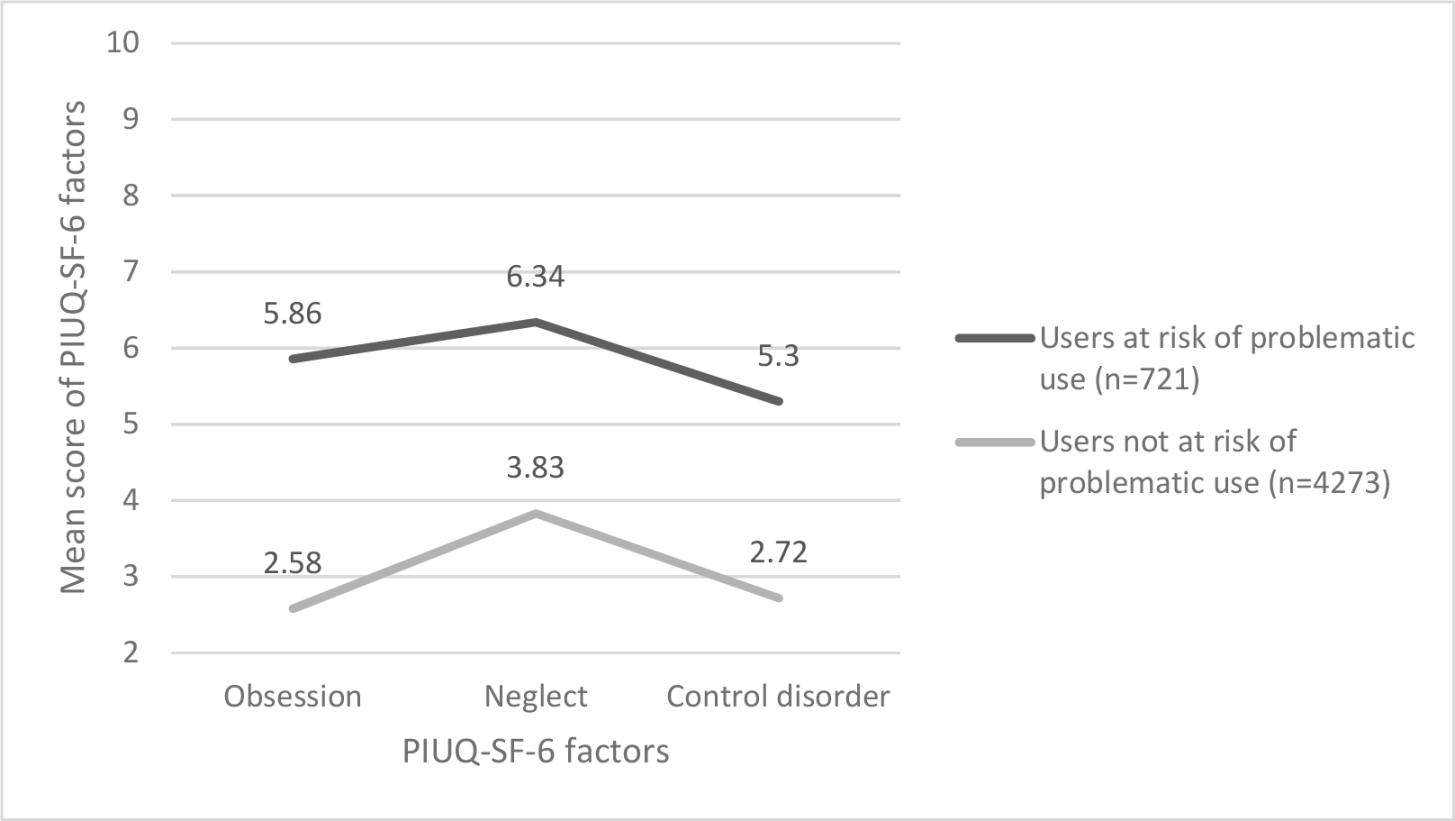
Latent profile analysis on the three factors of the Problematic Internet Use Questionnaire Short Form (PIUQ-SF-6) (N = 4994).

The mean scores of the two latent classes are presented in Fig 1. One of the two classes represents the majority of Internet users (85.56%) who are characterized by low scores on the three dimensions of the problematic Internet use scale. The mean of the total score on the PIUQ-SF-6 is 9.12 (SD = 2.41, 95% CI: [9.03–9.20]) for this group. The other class represents the minority of internet users (14.44%) who can be characterized by scores much above the average PIUQ-SF-6 score of the sample (i.e., 10.34 SD = 3.92). The mean of the total score on the PIUQ-SF-6 for this group is 17.62 (SD = 3.25, 95% CI: [17.34–17.89]) therefore they can be considered the at-risk group. In both groups the ‘neglect’ dimension showed an elevated level compared to the ‘obsession’ and ‘control’ disorder dimensions.

In order to test the concurrent and convergent validity of the scale, the two latent classes were compared using several variables relevant to the phenomenon of PIU. Users belonging to the at-risk class were more likely to (a) use the Internet for more than six hours in an average day, (b) have lower grade point average, (c) have lower self-esteem, and (d) have a higher level of depressive symptoms than rest of the users (Table 4). Among their three preferred online activities, at-risk users were also more likely to indicate playing online games, online chatting, and social networking, but less likely to indicate browsing the Internet for information and sending emails.

**Table 4:** Comparison of the two latent classes: testing equality for latent class predictors.

	No-risk class (n = 4273)	At-risk class (n = 721)	Overall test	
			Wald χ^2^	*p* value
Gender (male %)	51.3	48.0	2.39	.122
Age (years), mean (SE)	16.42 (0.014)	16.40 (0.033)	0.33	.567
Internet use in an average day (≥6 hours %)	19.1	40.0	105.33	< .001
Grade point average (min 10, max 50, mean 35.5; failed <20), Mean (SE)	34.31 (0.176)	33.34 (0.421)	4.43	.035
Self-esteem (min 10, max 40, mean 28.2); Mean (SE)	28.56 (0.083)	25.96 (0.217)	121.27	< .001
Level of depressive symptoms (min 6, max 24, mean 11.5); Mean (SE)	11.14 (0.050)	13.58 (0.141)	259.68	< .001
Top three Internet activities:				
Surfing, browsing, searching for information %	46.5	39.6	10.80	.001
Playing online games %	22.9	28.9	9.93	.002
Chatting, talking (e.g., MSN, Skype) %	67.1	76.5	25.92	< .001
Social networking (e.g., Facebook, Twitter) %	77.2	80.9	4.88	.027
Sending e-mails %	18.8	14.0	10.03	.002
Downloading (e.g., music, movies) %	58.0	57.3	0.10	.748

N = 4994.

### Recommended cut-off score to classify Internet users as users at-risk of problematic use

Until a clinical validation process would be carried out, a statistically established cut-off score for diagnosing those at-risk for problematic Internet use will have to suffice. Using the membership in the second class (i.e., being at-risk for problematic Internet use) as the “gold standard”, the sensitivity, specificity, PPV, NPV, and accuracy of the PIUQ-SF-6 at possible cut-off scores were calculated (see Table 5). Based on these values, a cut-off score of 15 (out of 24) is suggested as an optimal threshold to be classed as at-risk of problematic Internet use. In this case, specificity was 98%, while sensitivity was 85% meaning that 2% of the non-problematic cases are mistakenly considered as problematic and 15% of the true problematic cases are not recognized. Accuracy with this threshold is 96%, NPV is 98%, and PPV is 87%. Decreasing the cut-off score would lead to more false positive cases, whereas increasing it would result in more false negative cases.

**Table 5:** Calculation of cut-off thresholds for Problematic Internet Use Questionnaire Short Form (PIUQ-SF-6).

Cut-off	True positive	True negative	False positive	False negative	Sensitivity (%)	Specificity (%)	PPV (%)	NPV (%)	Accuracy (%)
12	706	3415	784	0	100	81	47	100	84
13	702	3755	444	4	99	89	61	100	91
14	672	3971	228	34	95	95	75	99	95
**15**	**603**	**4106**	**93**	**103**	**85**	**98**	**87**	**98**	**96**
16	504	4176	23	202	71	99	96	95	95
17	408	4198	1	298	58	100	100	93	94
18	313	4199	0	393	44	100	100	91	92
19	224	4199	0	482	32	100	100	90	90
20	165	4199	0	541	23	100	100	89	89
21	84	4199	0	622	12	100	100	87	87
22	59	4199	0	647	8	100	100	87	87

The bolded row in the table indicates the suggested cut-off threshold. N = 4905.

### Using PIUQ-SF-6 as a continuous scale

Although cut-off scores are particularly useful in several cases, by dichotomizing continuous scales, a big part of the information provided by these instruments is lost. Therefore, scales should be used in their continuous form, whenever possible. Table 6 presents the correlations between the PIUQ-SF-6 and some of the aforementioned predictor variables to provide additional information about the degree of associations of these variables with problematic internet use.

**Table 6:** Correlation between the Problematic Internet Use Questionnaire Short Form (PIUQ-SF-6) total score and predictor variables.

	Bivariate correlation	Corrected correlation^a^
Internet use in an average day	.36**	.47**
Grade point average	-.06**	-.08**
Self-esteem (α = .86)	.23**	.28**
Level of depressive symptoms (α = .82)	.34**	.43**

^a^ Corrected correlations were calculated using the Cronbach’s alpha values of the scales according to the following equation: r_corrAB_ = r_oiginalAB_/(α_A_ x α_B_)^1/2^ [24]

** *p* < .01.

## Discussion

The present study aimed to validate the 6-item version of the PIUQ on a representative national sample of adolescents. The results showed that the original three-factor model fitted the data adequately and factor loadings were conveniently high. Discriminant validity of the three-factor solution was further tested with MIMIC analysis showing that a considerable part of PIU related variables influenced the three factors differently. Latent profile analysis revealed 14.4% of the sample belonging to the at-risk group. This result is in line with previous findings where 18% of the adolescents were classified as belonging to the at-risk group [8]. Due to the lack of a gold standard, a cut-off value of 15 (out of 30) is recommended to differentiate between adolescents at-risk of problematic Internet use and the no-risk group, based on the two LPA classes and using a sensitivity and specificity analysis.

Nonetheless, the rate of those being at-risk is worryingly high. According to a recent methodological paper [25] the positive predictive value of screening instruments having average or even good sensitivity and specificity values (calculated using clinical studies) is surprisingly low when assessing addiction-like problematic behaviors with low prevalence rates. For instance an instrument having a sensitivity and specificity of 85%, has a positive predictive value of 9.24% if the true prevalence rate of the problematic behavior is 2%. This means that only 9.24% of those who scored positive on the test are truly problematic cases, the rest of the participants are labeled mistakenly. The low positive predictive values should be taken in consideration when interpreting the results of large-scale surveys (including the present one), because over-pathologizing everyday behaviors may have its harmful consequences [26]. Furthermore, due to the lack of a gold standard, it is increasingly important to validate future screening instruments using clinical samples.

The ‘neglect’ dimension showed an elevated level in both LPA classes. The two items of this dimension (“How often do you spend time online when you’d rather sleep?” and “How often do people in your life complain about spending too much time online?”) indicate excessive use of the Internet while not caring about their own sleep and that people around them notice and complain about the time they spend online. A previous study also showed an elevated level of neglect for at-risk adolescents compared to the ‘obsession’ dimension but not to the ‘control disorder’ dimension [8]. Consequently the ‘neglect’ aspect of PIU should be given special attention when developing prevention and treatment programs.

When reducing the number of items in the case of the PIUQ-SF-6, the aim was to preserve the original factor structure (including the names of the subscales) for future comparisons. However, in the case of the ‘obsession’ scale, the item reduction resulted in a content modification. The original subscale comprised seven items describing preoccupation (fantasizing, daydreaming about the internet when not online) and withdrawal symptoms. When selecting the final items of the shortened scale, two withdrawal items proved to be the most appropriate–although it was decided to leave the name of the subscale (i.e., ‘obsession’) unchanged.

Internet users belonging to the at-risk group were more likely to use the Internet for more than six hours in an average day, have a slightly lower grade point average, have lower self-esteem, and report a higher level of depressive symptoms than the rest of the Internet users. All these are in line with previous findings of the literature. Although spending increasing amounts of time online does not necessary indicate problematic Internet use, those who score high on PIU scales spend considerably more time online than users with low PIU scores [9, 27]. Previous research demonstrates that lower academic performance [28–30], lower self-esteem [31, 32], and higher level of depressive symptoms [28, 33–37] have been found to characterize those at-risk of PIU. No gender difference was found between the at-risk and no-risk groups in the present study. This is in line with some previous findings but contradicts many others. A review of 20 relevant studies by Carli and colleagues [33] reported only three studies found no gender differences, while eleven studies found significantly higher rates of PIU among males compared to females.

Despite the nationally representative sampling method and the large sample size, there are some limitations that should be noted. The self-reported nature of the data makes it vulnerable to biases (i.e., responses may have been influenced by biases in memory recall, social desirability, or the current affective state of the participant at the time of data collection). In addition, adolescent sample was from a single country (i.e., Hungary); therefore results should be generalized to other cultures with caution. Determining problematic users and calculating the suggested cut-off value based on a latent profile analysis instead of using an objective gold standard (i.e., clinically diagnosed group of problematic internet users) is also an important limitation of the present study. As noted above, future studies must validate screening instruments using clinical samples. The brevity of the instrument has both advantages and disadvantages. While brevity makes the 6-item PIUQ suitable for large-scale surveys assessing many different behaviors and/or constructs as well as targeting impulsive populations (e.g., adolescents), a limitation is that fewer items decreases the comprehensiveness of assessing the construct in-depth. Furthermore, since the subscales only comprise two items each, caution is warranted when using and interpreting them by themselves. However, overall, the 6-item version of the PIUQ appears to be appropriate for future research with the capacity to differentiate between Internet users at-risk of developing PIU and those not at-risk.

## Supporting Information

S1 Appendix Problematic Internet Use Questionnaire Short Form (PIUQ-SF-6). (DOCX)
