# Supplementary material for: Psychometric Properties of the Problematic Internet Use Questionnaire Short-Form (PIUQ-SF-6) in a Nationally Representative Sample of Adolescents
Source: PLoS One. 2016 Aug 9;11(8):e0159409. doi: 10.1371/journal.pone.0159409 (PMC4978438; doi:10.1371/journal.pone.0159409)
Supplement: S1 Appendix — (DOCX) [file pone.0159409.s001.docx]

**S1 Appendix. Problematic Internet Use Questionnaire Short Form (PIUQ-SF-6)**

In the following you will read statements about your Internet use. Please indicate on a scale from 1 to 5 how much these statements characterize you.

|  | never | rarely | some-times | often | always/ almost always |
| --- | --- | --- | --- | --- | --- |
| 1. How often do you spend time online when you’d rather sleep? | 1 | 2 | 3 | 4 | 5 |
| 2. How often do you feel tense, irritated, or stressed if you cannot use the Internet for as long as you want to? | 1 | 2 | 3 | 4 | 5 |
| 3. How often does it happen to you that you wish to decrease the amount of time spent online but you do not succeed? | 1 | 2 | 3 | 4 | 5 |
| 4. How often do you try to conceal the amount of time spent online? | 1 | 2 | 3 | 4 | 5 |
| 5. How often do people in your life complain about spending too much time online? | 1 | 2 | 3 | 4 | 5 |
| 6. How often does it happen to you that you feel depressed, moody, or nervous when you are not on the Internet and these feelings stop once you are back online? | 1 | 2 | 3 | 4 | 5 |

*Note:* Items belong to three subscales: Obsession (item 2 and 6), Neglect (item 1 and 5), Control disorder (item 3 and 4)

The PIUQ-SF-6 is available in multiple languages (i.e., French, Italian, Slovenian, Norwegian, Korean, Japanese, Persian, Czech, and Hungarian). For further information, please contact the corresponding author.
